# Prevalence of Different Kinds of Maxillofacial Fractures and Their Associated Factors Are Surveyed in Patients

**DOI:** 10.5539/gjhs.v6n7p66

**Published:** 2014-09-18

**Authors:** H. Latifi

**Affiliations:** 1 Department of Otolaryngology, Imam Khomeini Hospital, Faculty of Medicine, Reproductive Health Research Center, Urmia University of Medical Sciences, Urmia, Iran

**Keywords:** maxillofacial fractures, maxillary fractures, mandibular fractures, cheekbone fractures, dentoalveolar fractures, nasal fractures

## Abstract

**Introduction:**

Nowadays maxillofacial fractures have increased. In this study prevalence of different kinds of maxillofacial fractures and their associated factors are surveyed in patients referred to Imam Khomeini Hospital, Urmia in 2011.

**Methods:**

The study was across-sectional observational study. 637cases of patients with a confirmed diagnosis of maxillofacial fractures in 2011 referred to Imam Khomeini Hospital, Urmia and their data records were analyzed using SPSS software and chi-square tests.

**Results:**

In this study, 457 patients were male and 178 were female and the mean age was 14.47±26.68 years. Falling was the most common cause of fractures after accidents and assaults were the most common causes. The most common site of nasal fractures was about 66.4% and then fractures in several places about 14.9% and mandibular 7.1%.

**Conclusions:**

Based on the results obtained in the present study with other studies in this area it is concluded that maxillofacial fractures in males and in 20 to 30 years of age is prevalent and is mostly due to falling and road accidents and are further seen in nasal bone and mandible.

## 1. Introduction

Maxillofacial is the most important area of the body, starting from the base of the skull to the hyoid bone, this part of the body include the important organs of the body and bones (B.D. 1998). Spectrum of bone resistance of the bones of this part, changes from the weakest bone, cheek bone (Zmc) to the most resistant in the frontal bone, also the maxillofacial region is associated with systems such as visual, auditory, olfactory and speech and these two factors has expanded the range of injuries into these areas. Of social relationships, face is the most important and the first personal identity in personal relationships and so any damage to this region provides a kind of imbalance and mental health context. On the other hand, cultural factors, the amount of traffic and urban civilization today, all have influence on the increase or decrease of the trauma in this body site. The incidence of fractures will vary from one country to the other (Rowe & Williams, 1986). Mentioned reasons lead to increased attention and importance to the damage of the maxillofacial region (B.D., 1998). Injuries occur in this region, followed by many types of trauma, force to the bone in the elastic range causing the deformation and after force removal, bone returns to its previous state, but if the force be greater than the elasticity of bone, a permanent displacement and be irreversible. Entering force, energy is stored in the bones and while breaking energy as heat and tears of the bones and releases of the tissues surrounding and trauma occurs. The most common cause of trauma is from motor vehicle accidents, cars, crashes and fights etc. highest number of injuries and fractures of the maxillofacial region is related to the mandibular and nasal bone (Rowe & Williams, 1986). Given that the statistics of the incidence of maxillofacial fractures in Urmia not exist in the project we will address this issue. In this study we tried to answer the poor documentary data in the prevalence of fractures separately as the site of injury, age, se, cause of injury, the season of injury, etc. Increasing industrial appliances have increased the risk of bodily injury. In most accidents the injury is occurred to maxillofacial area. There are several epidemiological studies in many parts of the world about the frequency of facial trauma, injury causes and effects. The results of several studies suggest that this prevalence, causes, and the most common site of maxillofacial injuries varies in different countries and this rate is changing every year. The incidence of maxillofacial fractures in trauma patients has been reported from46.1%to74.4% (Zacharides, 1990). In Iran, Qazvin the most common cause of maxillofacial fractures in a fall from a height was 31.9% and crash was 23.3%. Removing the nose fractures of car accident 32.2%, the most common cause of maxillofacial fractures has been reported (Khorasani, 2007). In Hamadan in 2002 in patients admitted to Legal Medicine, the most frequent cause of facial fractures have been reported due to disputes involving 74.6% (Afzali & Ghaleiha, 2006).

The incidence of maxillofacial fractures was stated 74.4% in Jordan in 1998. Recent studies in the United Arab Emirates in 2007 indicated the prevalence of mandibular fractures 70.5%. In Japan, the most common cause of maxillofacial injuries is evaluated road accidents (52%) and the most common site is mandibular fracture (56.9%) (Lida et al., 2001). It seems one of the priorities of research in this part is to answer the question that the incidence of maxillofacial fractures percentage and what are the relevant factors that given the causes and related factors can determine a proper way to reduce the rate of prevalence of different fractures. The rate of 46.1%to74.4% showed a very high prevalence that will have much higher human and social costs. According to the above studies and apparent differences in the results and lack of doing research in these units need to research on determining the frequency of maxillofacial fractures and its related factors in patients referred to Imam Khomeini Hospital in 2011 to better plan in this area was felt and thus this cross-sectional study was performed to do so.

## 2. Methods

The study is across-sectional observational study on 637 cases of patients with diagnosis of maxillofacial fractures referred to Imam Khomeini Hospital in a one year time span (from the beginning of 2011 to the end of 2011) Samples with 95% confidence level, with alpha error 0.05, include 637 patients. All files available at this time as census were studied. The study was conducted as an observational cross-sectional study. In this study, patients referred to Imam Khomeini Hospital, Urmia in 2011because of the conflict, accidents, and falls from height etc. suffered injuries in the maxillofacial site were studied. Sample volume was conducted as census of the cases referred to Imam Khomeini Hospital, Urmia with a diagnosis of the maxillofacial site in 2011. Questionnaire was designed for this purpose. The first part of the questionnaire related to demographic data (age, sex, history of lesion), which according to records was completed. Epidemiological data and information forms the second part and the first paragraph was related to the cause of fracture that partners with regard to existing files specify the cause of them. Falling from height, motor accident, car accident, conflict, sporting events, dealing physical, damage by animals, accidents at work was predicted in the information form. If there are other factors, this factor against the other cases and with the exact cause of was brought. In the next section of the data form the fracture site with regard to the information was specified. Comparison of the prevalence of each fracture in terms of the cause of fracture and comparison of the prevalence of each fracture in terms of the role of each factor related to the fractures were evaluated using statistical tests (chi-square and ANOVA). Finally, after the data were collected using a questionnaire designed by the researcher (see appendix), took action to review the data analysis that was performed using SPSS software (Dalfard et al., 2013). The results were presented in the form of tables and charts with classification of maxillofacial and nasal fractures based on age, sex, fracture cause and symptoms of the fracture.

## 3. Results

This study is across-sectional study during 2011. Study was performed on 637 patients referred to the department of oral and maxillofacial surgery of Imam Khomeini Hospital. The mean age of patients participating in the study was 14.47±26.68 years. Among the 637 patients, 457 patients (72%) were male and 178 patients (28%) were female. According to the results of Table (1) the frequency of patients with maxillofacial surgery referred to maxillofacial department is 2.7. In the second quarter were 182 (26.6%), in the third quarterwere180 (28.3%), in the first quarter 4.5 (23.6%) and in the fourth quarter 124 (19.5%) have been reported.

**Table 1:** Frequency of patients referred to maxillofacial surgery

	Frequency	Percentage
Three months 1	150	23.6
Three months 2	182	28.6
Three months 3	180	28.3
Three months 4	124	19.5

Total	636	100.0

According to the results in Table 2 prevalent fractures are as follows: Nasal 422 cases (66.4%), multiple 95cases (14.9%), mandible 45 cases (7.1%), orbital 23 cases (3.6%), zygomatic 21 cases (3.3%) and maxilla 19 cases (3%).

**Table 2:** Frequency of fracture

		Frequency	Percentage
Fracture site	Orbital	23	3.6
	Nasal	422	66.4
	Zygomatic	21	3.3
	Maxilla	19	3.0
	Mandible	45	7.1
	Multiple	95	14.9
	Total	636	100.0

According to information contained in Table 3: 223 cases (35.1%) of patients referred to the department of oral and maxillofacial surgery with a fall from a height and 166% cases (26.1%) due to accidents, 109 cases (17.2%) with no reason given, and 66 cases (10.4%) due to the conflict, 36 cases (5.7%) due to collision with an object,19 cases (3%) due to damage by animals, 8 cases (1.3%) due to accident at work, 3 cases (0.5%) due to exercise and 5 cases (18%) due to other causes were admitted.

**Table 3:** Fracture cause frequency

		Frequency	Percentage
Fracture cause	Falling from height	223	35.1
	Accidents	166	26.1
	Conflict	66	10.4
	Collision with an object	36	5.7
	No reason given	109	17.2
	Damage by animals	19	3.0
	Other cases	5	.8
	Exercise	3	.5
	Damage at work	8	1.3
	Total	635	100

Total	636		

166 cases referred to the department of oral and maxillofacial surgery due to accident disorder. 94 cases (57%) car accidents, 32 cases (19.4%) motorcycles and 28 cases (17%) as a pedestrian, 7 cases (4.2%) bike ride and 4 cases (2.4%) were in crashes with buses and heavy vehicles.

According to the results in Table 5, 17 cases (3.7%) male with an orbital fracture, 292 cases (63.9%) with a nasal fracture, 17 cases (3.7%) with zygomatic fractures, 16 cases (3.5%) maxilla, 35 cases (7.7%) mandible, 6 cases (1.3%) facial, 74 cases (16.2%) multiple had been admitted in maxillofacial surgery unit, Imam Khomeini Hospital.

**Table 4:** Frequency of accident types

		Frequency	Percentage
Accident type	Pedestrian	28	17.0
	Bicycle	7	4.2
	Motorcycle	32	19.4
	Car	94	57.0
	Bus and heavy vehicle	4	2.4
	Total	165	100.0

Total	636		

**Table 5:** The frequency of fracture site according to sex

			Fracture site	Total					

			Orbital	nasal	Zygomatic	Maxilla	Mandible	Multiple	
Male		Count	17	292	17	16	35	74	451
		% within sex	3.7%	63.9%	3.7%	3.5%	7.7%	16.2%	
	Female	Count	6	130	4	3	10	20	173
		% within sex	3.4%	73.0%	2.2%	1.7%	5.6%	11.2%	
Total	Count	23	422	21	19	45	94	635	
	% within sex	3.6%	66.5%	3.3%	3.0%	7.1%	14.8%		

In female, 6 cases (3.4%) were orbital, 130 cases (73%) nasal and 4 cases (2.2%) zygomatic, 3 cases (1.7%) maxillary, 10 cases (5.6%) mandibular and5 cases (2.8) facial and 20 cases (11.2%) multiple.

According to the results of the Pearson chi-square statistic P-Value equal to 0.208 and is more than 0.05 value, it is concluded that there is no significant relationship between gender and fracture in this study. According to the results shown in Table 6, in male the frequency of fracture fell from a height was 136 cases (29.8%), in accidents 124 cases (27.1%), conflict 60 cases (13.1%), collision with an object 29 cases (6.3%), damage by animals 16 cases (3.5%), no reasons given 78 cases (17.1%), exercise 2 cases (0.4%), accident at work 8 cases (1.8%) and 4 cases (0.9%) account for other cases.

**Table 6:** Frequency of fracture cause according to sex

		Fracture cause	Total								

		Falling	Accidents	Conflict	Collision	No reason given	Damage from animal	Other	Exercise	At work	
Male	Count	136	124	60	29	78	16	4	2	8	457
	%within sex	29.8%	27.1%	13.1%	6.3%	17.1%	3.5%	.9%	.4%	1.8%	100.0%
Female	Count	87	41	6	7	31	3	1	1	0	177
	%within sex	49.2%	23.2%	3.4%	4.0%	17.5%	1.7%	.6%	.6%	.0%	100.0%
Total	Count	223	165	66	36	109	19	5	3	8	634
	%within sex	35.2%	26.0%	10.4%	5.7%	17.2%	3.0%	.8%	.5%	1.3%	100.0%

In women, the prevalence of fractures in the fall was 87 cases (49.2%), traffic accidents 41 cases (23.2%) conflict 6 cases (3.4%), collision 7 cases (4%), injury 3 cases (1.7%) no reason given 31 cases (17.5%) exercise 1 cases (0.6%) at work (0%) other 1 case (0.1%).

According to the results of Pearson chi-square statistic value of p-value was equal to 0.000 that is less than 0.05. There is a significant relationship between gender and fracture. In men, the most common cause was falls from height by 29.8% and in women the most common cause was falls from a height by 49.2% that in women falling from height was greater than that of men.

## 4. Discussion

The results of this study showed that car accidents and falls from height were the most common causes of maxillofacial fractures more over nasal and mandibular also formed the most damaged parts. The age and gender of patients and also injuries with type of injury significant correlation was not observed (P>0.05).

## 5. Compare the Results With Similar Studies

A study in Japan by Sugiura et al. about the incidence of fractures of the maxillofacial region between 1981–1996 AD years on 1502 patient records (with available data) was performed. In this study the prevalence in males was 2.8 than females and the most commonage-related damage was 10-29 years. However, in our study, the male to female ratio was close to 2.7. Results of the study indicate the most common cause of maxillofacial injury, road accidents (52%) and the most common site of fracture, mandibular (6.9%) (Sugiura T. 2001), in the case of road accidents, we achieve the same results; but the most common site of injury in our study was nose. Adebayo et al. study in 2003 in Nigeria, the prevalence and forms of damage to maxillofacial in Kadvana state of the country were surveyed. Therefore, 443 patients ranging from 1991 to 2000 AD with the reasons of maxillofacial injuries were referred to teaching hospitals of Kadvana, in terms of the prevalence of trauma in Victoria from July 2001 until the end of 2004 were examined. Collected data included demographic data and their need to have maxillofacial surgery. Results showed that the majority of trauma patients were with traumatic maxillofacial region and the most common prevalence was in the age range15-25 years, which is in direct line with the findings of our study. Involvement of ratio of male to female was 3 to 1 that were lower than figures reported in our study. Traumatic injuries are often caused by accidents that are consistent with our study, but common fractures were associated with fractures of the upper jaw (Shahim et al., 2006), which our results are not the same. Eggenspeger et al. study in 2006 in Switzerland frequency of maxillofacial injury due to occupational accidents was studied in a 3-year monitoring. The study was conducted in main hospital services related to oral and maxillofacial surgery of Bern for 2 consecutive years as prospective. Information about the causes of maxillofacial injuries, a common site of fractures and demographic data were collected. The results showed that the mean age of patients was 44 years and male to female ratio (with a maxillofacial injury depending on the job) was 4 to1 that are not similar to our study, the mean age was lower in our study. Most occupational injuries caused by falling objects and collisions with accessories. 82% of fractures like our study were in maxillofacial site that in 69% of cases there is a need for surgery (Eggensperger & Danz,. 2006). Dr. Nosrati et al.in a study in 2005 examined the epidemiology and mandibular fractures in Bu-Ali Sina, Shafa and Nime- Shaban Hospitals, Sari, records of patients were studied with fractures during 9 months from August 2005 to April 2006 in the reception centers. In this cross-sectional study, 125patients (106 male and 19 female) aged between 2 to 79 years in terms of age, sex, cause and location of the lower fractures were studied. The most affected age decade was from 20 to 29 years and the incidence of mandibular fractures in males was 5.5 to females, the most common mechanism of fracture in55% of cases was vehicle accidents and the most common site of mandibular fracture was trunk (27%), premphase (24.7%%) and the mandibular angle (18.5%), respectively (15), all these findings, including age and sex ratio conflict is somewhat similar to our study. In addition, the location and mechanism of injury is similar to our study (Nosrati et al., 2012).

In a study by Dr. Afzaland and Dr. Ghaleiha in 2002 on epidemiological study of assaults and injuries in Legal Medicine Center in Hamadan was performed in 2002, 9828 patients with trauma by different reasons were referred as outpatient and were referred to Legal Medicine Center, Hamadan, were studied. Who are referred by the police or a judicial authority had been studied by practitioners of that center and lesions were collected in specific forms of information. Then by the physician’s discretion in terms of lesions, those who need re-examination in given time spans were examined and the process would continue until recovery of lesions or impairment or death. The results showed that 76% of patients were male and 24% were female, like our study male ratio is higher. Most referred ages by the number of 3218 people were between 20-29 years with a mean age of 26 ± 4.7 years are consistent with our study. The most common cause (74.6%) was quarrel and in terms of the cause and motive of creating mayhem in the majority of cases (43.5%) was unknown. The most common devices were hard objects (91.2%) in 43.5% of the cases, most times were between 12-18 hours. The highest number of patients (32.3%) was in summer, especially in May (11.4%). The most common lesion was bruising on the body surface (46.5%) in 99.4% of patients achieved complete remission and only 0.6% died due to injuries and complications (Afzali & Ghaleiha, 2006). However, in our study, despite the mentioned study falling and road accidents were far higher. Al-khateeb et al, study in 2007 on the frequency of maxillofacial injuries in the three major hospitals in the UAE, the records of 288 patients with maxillofacial injuries were examined. The age range of patients with maxillofacial injuries had been reported in the range of 2 to 82 years that male to female ratio was 7 to 4 which were similar to those obtained in our study. The frequency of maxillofacial injuries was maximum in January, and most patients had mandibular fractures (70.5%). Frequency of maxillofacial injuries were reported in this studyabout36% that second records of 518 patients with fractures of the maxillofacial region, in the years 1994 to 2004 were evaluated with available data. The results show that the most prevalent fracture site was mandibular bones (46.1%) and the main reason for fractures was attributable to falls from height and strife that causes fracture is somewhat similar to our study, but the study of nasal complex fracture was the most common site of injury. In maxillofacial bone fracture the major cause was conflict, but in the upper and lower jaw fractures, daily activities and occupational factors constitute the major cause (Al-khateeb et al., 2007).

Dr. Mahmoud Hashemi et al. study in 2008 was conducted to assess the etiologic association with the type and location of mandibular fracture in patients referred to Shariati Hospital, Tehran, in across-sectional study on 358 patients with mandibular fracture were referred to the center of the Oral & Maxillofacial Surgery, Shariati Hospital, Tehran, information of the patients were recorded in the questionnaire included age, sex, fracture time, fracture site and associated fractures. Of these, 192 cases were with complex fracture (56%), 124 cases with simple fractures (30.5%) and 42 cases (10.5%) were associated with fractures of other bones. Among referrals 82.9% was males and 17.1% was females that are the evidence that men are more at risk because of their job (Hashemi H. 2008). The sex ratio observed in this study is somewhat similar to our findings. Ina study conducted by Barros de et al. that the results published in 2010 in the form of a paper was announced that maxillofacial fractures despite a high prevalence taking appropriate measures such as the use of seat belts are preventable and therefore considering a for prevention can prevent additional costs (Barros, Campolomgo, Zanluqui, & Duarte, 2010). The subjects are applicable in our study given that accidents constituted the most maxillofacial fractures. In a study conducted by Chrcanovic et al. in Brazil that the results are published in 2010, 122 patients aged over 60 years were studied and it was found that falling is the main cause of maxillofacial fractures in this age group. The most bone fractures were of mandibular (Chrcanovic, Souza, Freire-Maia, & Abreu, 2010). However, in our study, which was conducted in all age ranges, falling is in the third rate of maxillofacial fractures that is quite different with our study. Ina study conducted by Yamamoto et al. in Japan that the results are published in 2010, with the study of 279 patients with maxillofacial trauma due to falls was announced that most of the subjects were males and frequent bone fractures was maxillofacial bone (Yamamoto et al., 2010). In our study, the prevalence was higher in males than in females, but nasal bones were more fractured.

## 6. Conclusions

In conclusion, according the results and their comparison with other studies in this field it is concluded that maxillofacial injuries in males and in 20 to 30 years of age were more prevalent and are mostly due to falling from height and road accidents and are observed mostly in nasal and mandibular bones.

## 7. Suggestions

It should be tried to reduce by public awareness, road accidents as the most common cause of maxillofacial fractures, including an emphasis on the use of safety belts and cars with air bags in reducing maxillofacial trauma.
